# Active immunotherapy, ACI‐24.060, induces anti‐Abeta antibodies with binding profiles mirroring clinically validated monoclonal antibodies

**DOI:** 10.1002/alz.089036

**Published:** 2025-01-09

**Authors:** Emma Fiorini, Chiara Babolin, Rakel Carpintero, Stefania Rigotti, Stefanie Siegert, Catherine Morici, Harishini Balachandiran, Jonathan Wagg, Nicolas Fournier, Lara Herriott, Piergiorgio Donati, Saskia Delpretti, Nuno Mendonca, Andrea Pfeifer, Madiha Derouazi, Marie Kosco‐Vilbois, Marija Vukicevic

**Affiliations:** ^1^ AC Immune SA, Lausanne Switzerland; ^2^ University of Oxford, Oxford United Kingdom; ^3^ AC Immune SA, Lausanne, Vaud Switzerland

## Abstract

**Background:**

The key advantage of active immunization is the induction of sustained, polyclonal antibody responses that are readily boosted by occasional immunizations. Recent clinical trial outcomes for monoclonal antibodies lecanemab and donanemab, establish the relevance of targeting pathological Abeta for clearing amyloid plaques in Alzheimer’s disease. ACI‐24.060 is a liposomal anti‐Abeta vaccine, using Abeta 1‐15 as the immunogen. In non‐human primates (NHPs), ACI‐24.060 induced antibodies binding to pathological Abeta species: 1) pyroglutamate, target of donanemab and 2) oligomers, with a high preference versus monomers, similar to lecanemab, suggesting a potential for Abeta clearance in humans.

**Method:**

Cynomolgus monkeys were immunized monthly with ACI‐24.060 (generated using the SupraAntigen® platform). Immune sera obtained after seven immunizations were tested using a competitive inhibition assay involving preincubation of immune sera or lecanemab with Abeta monomers or oligomers. Binding profiles to pyroglutamate‐Abeta and Abeta oligomers were established for vaccine‐induced antibodies, lecanemab, and donanemab, using ELISA‐based assays. Published lecanemab and donanemab exposure‐response models were utilized to simulate Amyloid‐PET lowering profiles for antibodies evoked by ACI‐24.060.

**Result:**

Immunization of NHPs with ACI‐24.060 induced a sustained polyclonal response with strong binding to Abeta toxic species, i.e. pyroglutamate‐Abeta and oligomers. The competitive inhibition assay demonstrated that the antibodies generated by active immunization of NHPs with ACI‐24.060 gave a 3‐log preferential binding for oligomeric over monomeric Abeta, similar to the differential observed with lecanemab. Binding activity of the vaccine‐induced antibodies to pyroglutamate‐Abeta and Abeta oligomers was in the range of levels of donanemab (11‐48 µg/mL) and lecanemab (18‐26 µg/mL), which are relevant for Abeta plaque clearance in human AD patients. Exposure‐response modelling in these ranges of ACI‐24.060‐induced antibodies demonstrated a potential for Abeta clearance in humans.

**Conclusion:**

ACI‐24.060 induced antibody responses in NHPs with 3‐log preferential binding to oligomeric versus monomeric Abeta, similar to lecanemab and strong binding to both Abeta oligomers and pyroglutamate‐Abeta species. Induced antibodies were in the range of levels of donanemab and lecanemab relevant for Abeta clearance in human AD patients. Therefore, ACI‐24.060, with its dual target effect (pyroglutamate‐Abeta and Abeta oligomers) and its FDA Fast Track designation, represents a potential breakthrough active immunotherapy for Alzheimer’s disease.

